# Mid-and long-term efficacy of endovascular-based procedures for Cockett syndrome

**DOI:** 10.1038/s41598-018-29756-1

**Published:** 2018-08-14

**Authors:** Jiasheng Xu, Yujun Liu, Weimin Zhou

**Affiliations:** 1 Department of Vascular surgery, the Second Affiliated Hospital of Nanchang University, No.1 Minde Road, Nanchang, 330006 Jiangxi Province China; 2 Graduate School of Nanchang University, Nanchang, 330006 Jiangxi Province China; 3 Queen Mary College of Nanchang University, No. 1 Minde Road, Nanchang, 330006 Jiangxi Province China

## Abstract

To investigate the mid- and long-term results of endovascular-based procedures for Cockett syndrome. The clinical data of 412 patients with Cockett syndrome treated between January 2003 and September 2017 were retrospectively analyzed. In these patients, 231 cases were acute left iliac femoral vein thrombosis (group A), and 181 cases were chronic venous insufficiency (group B), and different endovascular procedures and/or hybrid procedures were performed. In group A, the technique success rate was 100% (231/231); the left iliac vein in 5 patients showed no stenosis or occlusion, and the incidence of pathological changes in the left iliac vein was 97.8% (226/231); a total of 182 stents were implanted. In group B, the technique success rate was 99.4% (180/181); the average pressure difference between the proximal and distal portion of the pathological left iliac veins decreased from preoperative (18 ± 4.45) cmH2O to postoperative (4 ± 3.02) cmH2O (*P* < 0.01); 89 patients, complicated with valvular incompetence in the left superficial femoral vein, underwent a second-stage femoral valve repair. Follow-up ranged from 3 months to 8 years, with an average of 35.6 months, and intrastent thrombosis occurred in 15 cases of group A and in 2 cases of group B. Endovascular-based procedures offer favorable mid- and long-term results in treatment of Cockett syndrome, which in combination with Fogarty catheter thrombectomy or catheter-directed thrombolysis is a beneficial complementary treatment for patients with acute iliac femoral vein thrombosis.

## Introduction

Cockett syndrome, also known as iliac vein compression syndrome, is caused by the compression of right common iliac artery on the left common iliac vein, resulting in edema, varicose veins, chronic ulcers, deep venous thrombosis and so on. In the absence of early and effective treatment, Cockett syndrome can cause deep venous thrombosis of the left lower extremity^1^, destroy the venous valve of the lower extremity, affect the venous function of the lower extremity and lead to fatal pulmonary embolism. Lower extremity deep venous angiography and femoral vein catheterization are the gold standard for diagnosis of Cockett syndrome. Traditional methods of treatment is surgical revascularization, mainly including the following: Femoral-femoral venous bypass(Palma-Dale surgery), EPTFE artificial vascular graft, iliac vein angioplasty and artificial vascular graft with support ring^2–5^. These surgical procedures have resulted in greater trauma, more complications and a lower rate of vascular patency in the postoperative three years, and are gradually replaced by endovascular interventional surgery. Long term follow-up results of Cockett syndrome treated by endovascular technique in our center from January 2003 to September 2017 were summarized as follows.

## Data and Methods

### General information

412 patients with Cockett syndrome in our center from January 2003 to September 2017, including 187 males and 225 females, aged 22~76 years, with an average age of (43.5) years old, were included in this study. The stenosis or occlusion of left iliac vein were confirmed by surgery and color Doppler ultrasound and/or venography in all patient, with or without left iliac femoral popliteal vein thrombosis. All patients were divided into two groups, including A group of 231 cases of acute left iliac vein thrombosis where the course of disease was less than 7 d. In group B, 181 cases had chronic venous reflux obstruction; the course of disease ranged from 3 months to 14 years, with an average of 22 months. There was no difference in the general data of the two groups(Table 1). All patients had one or more of the following symptoms: lower limb swelling, pain, venous claudication, superficial varicose veins, chronic ulcers with or without femoral swelling. Conservative treatment (such as systemic anticoagulation, limb lifting, etc.) failed.

**Table 1:** Comparison of General data of patients.

Group	sex		age	hospital stay
	male	female		
A group	106	125	42 ± 3.5	6 ± 2.5
B group	81	100	44 ± 2.5	5 ± 1.5
P value	>0.05		>0.05	>0.05

The following cases were excluded from this study: chronic inferior vena cava and/or double iliac venous thrombosis, metastatic or primary pelvic malignant tumors, pelvic radiotherapy, iodine contrast agent allergy, renal failure, bleeding disorders, contraindications to anticoagulants, pregnancy or delivery period, abdominal or orthopedic surgery within 7d or hemorrhagic stroke within 1 year.

## Ethical Review Statement

### Approval

The study was approved by the ethics committee of the Second Affiliated Hospital of Nanchang University.

### Accordance

Authors of this article solemnly declares that the methods were carried out in accordance with the relevant guidelines and regulations.

### Informed consent (for experiments involving humans or human tissue samples)

Informed consent was obtained from all individual participants included in the study. All participants in the study voluntarily signed informed consent.

The study obtained informed consent for the publication of identifying information or images in an online open-access publication(when applicable).

### Treatment

#### vena cava filter placement, femoral vein incision thrombectomy (or femoral vein catheter thrombolysis), iliac vein balloon angioplasty and/or stent implantation

**Figure 1:**
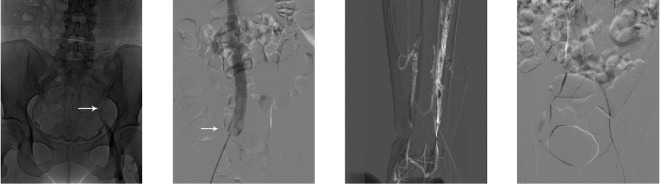
Vena cava filter placement and catheter-directed thrombolysis for acute iliofemoral vein thrombosis. (**A**) Deep vein angiography showing the fi lling defect in the left iliac vein. (**B**) Vena cava fi lter placement in the inferior vena cava below the renal vein. (**C**) Roadmap anterior tibial vein puncture. (**D**) 5-F Unifuse catheter placement in the iliofemoral vein.

**Figure 2:**
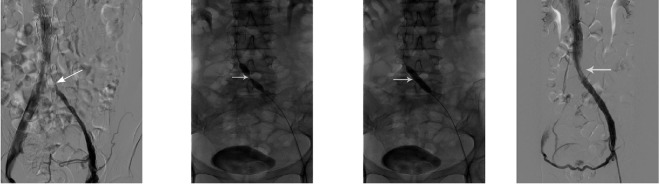
Balloon dilatation of the left iliac vein and stent placement. (**A**) Dissolution of the thrombus in the distal part of the left iliac vein, and presence of the fi lling defect of the left iliac vein (showed by arrow). (**B**,**C**) Balloon dilatation of the left liac vein. (**D**) Restoration of patency of the left iliac vein after stent implantation.

All patients in A groups accepted spinal anesthesia or local anesthesia by strengthening, vena cava filter, limb femoral vein incision of Fogarty balloon catheter thrombectomy (or iliac femoral venous catheter thrombolysis), lesions of iliac vein balloon angioplasty and/or stent implantation. Adjuvant therapy of anticoagulation, thrombolysis and antiplatelet therapy was performed after operation. First, the femoral vein puncture was performed by Seldinger technique on the uninjured side. Digital subtraction angiography was performed to determine the location of the renal vein opening, whether or not the inferior vena cava had malformation, variation or obstruction, and to measure the diameter of the inferior vena cava. The vena cava filter was implanted in the inferior vena cava of 0.5~1 cm below the opening of the renal vein. Make a longitudinal incision about 5~8 cm in the ipsilateral inguinal, exposure and free femoral vein 3~5 cm, block the proximal and distal femoral vein and make a transverse incision of the anterior wall. The 7 F Fogarty balloon catheter was inserted into the common iliac vein to remove the thrombus. Distal venous thrombosis is usually removed by beat extrusion. Using urokinase(Minim pump infusion)to dissolve thrombus(n = 74)(Unifuse, Angiodymic, USA)in the iliofemoral venous catheter by the approach of anterior tibial vein, posterior tibial vein, popliteal vein or small saphenous vein Since October 2011**(**Fig. 1**)**. The dosage of urokinase was 60~100 000 U/d, and the dosage and speed of drug administration were adjusted according to the 4 items of blood coagulation. The curative effect was observed every 2 days; the longest was not more than 7 days. Iliac femoral popliteal vein angiography confirmed no residual thrombosis in vein. Inserting the vertebral artery catheter and guide wire, leaving the guide wire into the inferior vena cava and taking out the catheter under the guidance of the catheter. The location of iliac vein lesions was dilated with a diameter of 12~16 mm balloon catheter, and the stent was implanted in the patients with residual stenosis **(**Fig. 2**)**.

The self-expanding metal stent was chosen, with diameter of 14~20 mm and length of 8~12 cm. Low molecular weight heparin sodium was given before the stent implantation, and a suitable stent was selected (15–20% for general enlargement of the diameter of the vessel), and the stent delivery system was introduced under Roadmap. The front end of the stent was generally placed around 2–3 mm in the inferior vena cava. After successful balloon dilatation or stent implantation, a further examination is needed to further examine the vascularity of the left iliac vein and the flow rate of the contrast agent into the inferior vena cava. If there are still strictures on the left iliac vein or the contrast agent can enter the inferior vena cava indirectly through the collateral circulation, it is necessary to consider if a larger saccule is needed to dilate the vessel again and whether it is necessary to implant the stent again.

Postoperative limb elevation of 30 degrees and subcutaneous injection of low molecular weight heparin sodium 40 mg in every 12 hours were performed. After 3 days, it is replaced with oral anticoagulant (warfarin sodium) for 6~12 months to ensure that the international normalized ratio is between 2.0~2.5. The affected limb was treated with a circulatory drive instrument for one week(6 h/d)and then replaced with a medical elastic sock for 3~6 months to promote blood flow, dissolve residual thrombus and prevent thrombus recurrence. The size of legs was measured every 2~3 days after operation to observe the curative effect.

#### Iliac vein balloon angioplasty and/or stent implantation

All patients in group B received Seldinger technique to insert the catheter sheath into left femoral vein under local anesthesia. First, the super soft guide wire passed through the lesion site of the left iliac vein. If it failed, the vertebral artery catheter or Cobra catheter can be used to guide the catheter through the inferior vena cava under the guidance of roadmap. The guidewire was inserted into the balloon catheter to dilate the lesion site of iliac vein. The dilation time was 1~3 minutes and the dilation pressure was 1~2 atmospheres (1 atmosphere = 101.325 kPa), repeated 2~3 times. If elastic retraction or residual stenosis occurs, the stent should be implanted, and the choice of stent is the same as that in group A. The diameter of the stent should be 10~15% larger than the diameter of the normal iliac vein, so as to prevent the stent displacement and make it continue to expand. The pressure difference between the proximal and distal ends of iliac vein lesions was measured before and after operation. For the patients with moderate or severe blood flow in the first pair of valves on the left femoral superficial vein, the femoral superficial vein was repaired after successful endovascular treatment of 3~5 d^6^. After the surgery, wear medical elastic stockings for half a year and take oral warfarin sodium for 6~12 months, to ensure the international normalized ratio between 2.0~2.5.

### Statistical processing

The data were analyzed by mean ± standard deviation ($\overline{{\rm{x}}}$ ± S). SPSS 18.0 statistical software was used for analysis. The T test was used to compare the pressure difference between the two groups before and after operation.

## Results

### Surgical results

The technical success rate of 231 patients in group A was 100% (231/231); 5 of them had no stenosis or occlusion of the left common iliac vein, and the incidence of left iliac vein disease was 97.8% (226/231). Stent was placed in 59 patients with residual stenosis in group A1 during operation (Table 2). This group of 182 stents were implanted in 1880 [COOK Z type 42 stents, Wallstent (Bostonscientific, USA) 56, Lumineex (Bard, USA) 58, Optimed Sinus 26]; implantation of inferior vena cava filter [231 pieces of home-made Xianjian vena cava filter 71, TrapEase (Cordis, USA or Optease 103) and Vena Tech (BRAUN, France) 57], the temporary filter in 98, 133 permanent vcf.

**Table 2:** Subgroup and stent placement in group A.

Group A	technology	number	stent implantation/ residual stenosis	PTS	Postoperative restenosis
Group A1	balloon angioplasty and/or stent implantation	152	123	3	3
Group A2	urokinase dissolution of thrombi + balloon angioplasty and/or stent implantation	74	59	2	0
*P* value			>0.05		

The technical success rate of 181 patients in group B was 99.4% (180/181). 151 stents were implanted in this group[COOK 1880 Z stent 20, Wallstent 41, Lumineex 37, Optimed Sinus 24, Protege(EV3, USA) 29] (Table 3); The super soft guide wire failed to pass through the lesion site of the left iliac vein in 51 patients, and the catheter was placed in the inferior vena cava under the guidance of roadmap. (The rate of failure of guidance of the super soft guide wire through the lesion site of the left iliac vein was 32.28%) In 1 cases of B group, the rupture of the left iliac vein resulted in huge retroperitoneal hematoma and hemorrhagic shock during the balloon dilation of the iliac vein, which was improved after active rescue.

**Table 3:** The type of stent used in the two groups.

Group/stent type	Technical success rate	COOK 1880 Z	Wallstent	Lum ineex	Optimed Sinus	Protege
A group	100%	42	56	58	26	0
B group	99.40%	20	41	37	24	29

In group B, the pressure difference between the proximal and distal ends of iliac vein lesions was (18 ± 4.45) cmH2O (1 cmH2O = 0.098 kPa) before the operation, and the postoperative pressure difference was (4 ± 3.02)cmH2O (*P* < 0.01). In group B, 89 patients had severe valvular insufficiency of left superficial femoral vein, and underwent second stage valve repair.

### Follow up results

All patients were followed up by color Doppler ultrasonography or venography for 3 months to 8 years, with an average of 35.6 months. The quality of life was assessed by telephone or communication and the diagnosis of post thrombotic syndrome was performed.

195 of 231 patients in group A were followed up, with a follow-up rate of 84.4% (Table 4). Among them, 183 cases were cured, the clinical cure rate was 93.8% (183/195), and 12 cases improved. The follow-up period ranged from 3 months to 8 years, with an average of 2.8 years. 182 cases of patients with iliac vein stent implantation, which followed up for more than 6 months in 128 cases, 15 cases of stent thrombosis; In 49 cases without stent implantation, 40 cases were followed up for more than 6 months, only 3 cases had iliac vein occlusion. Post thrombotic syndrome occurred in 5 patients, and the incidence of post thrombotic syndrome was 2.6% (5/195).

**Table 4:** Follow-up results.

group/value	stents implanted	followed up	Follow-up rate	Clinical cure rate	Mean follow-up time (years)	PTS	Postoperative restenosis
A group	182	195	84.40%	93.80%	2.8 ± 0.16	5	3
B group	151	158	87.30%	100%	2.2 ± 0.68	2	0

PTS: Post thrombotic syndrome.

181 patients in group B were followed up, with a follow-up rate of 87.3%. The follow-up period ranged from 3 months to 8 years, with an average of 2.2 years. The clinical cure rate was 100% (120/120). 151 cases were implanted iliac vein stent. 128 cases were followed up for more than 6 months, thrombosis occurred in the stent in 2 cases. There were 30 cases without stent implantation, 20 cases (including 1 unsuccessful cases) who were followed up for more than 6 months, and no iliac vein thrombosis occurred.

### Complications and Management

The main postoperative complications were thrombosis recurrence (n = 17), contralateral deep vein thrombosis (n = 2), inferior vena cava filter thrombosis (n = 12) and stent thrombosis (n = 17); all of them were treated conservatively. One case of iliac vein rupture, hemorrhagic shock and retroperitoneal hematoma was improved by conservative treatment. Other complications included puncture site (n = 3) or incision (n = 2) hematoma formation, incision bleeding (n = 1) and cerebral hemorrhage (n = 1). Except for 1 case who underwent reoperation because of bleeding within the incision, the rest were treated conservatively. There were no deaths due to pulmonary embolism in this group.

## Discussion

Cockett syndrome is also known as May-Thurner syndrome or iliac vein compression syndrome. As early as 1851, Virchow published his preliminary observations, but the knowledge of the disease was not enough at that time. It was not until 1908 that McMurrich found Spinous substance (spurs) in the common iliac vein and considered as a congenital anomaly”. May and Thurner In 1957, Cockett and Thomas^7^ in 1965 both of them described the disease in detail. It was considered that the compression to the left iliac artery caused by the right iliac vein and pulsatile injury caused by the contact of the vein resulted in the vein adhesion or the formation of spines. It is generally believed that once right iliac artery bestride the left iliac vein^8–10^, either lesions in or out of the cavity of the left common iliac vein caused complete or incomplete obstruction resulted in the corresponding symptoms are called Cockett syndrome^11^.

Venography is the first choice in the diagnosis of Cockett syndrome. Venography can clearly show the filling or defect of the left common iliac vein, the expansion of the distal iliac vein, the slow flow of the venous blood flow, and the formation of the collateral vessels of the pelvis^12^. The diagnostic accuracy of color Doppler ultrasound in Cockett syndrome is limited, because it is affected by the bladder, intestinal cavity and fat, but it is more accurate in the diagnosis of iliac vein thrombosis^13^. Computed tomography angiography, magnetic resonance angiography, computed tomography venography, magnetic resonance venography as noninvasive examination is helpful for the diagnosis of iliac vein stenosis or occlusion, and it can identity the compression from the right iliac artery^14^, but it is more expensive, cannot be widely used. In addition, we also found that the left iliac vein was compressed by the right iliac artery^15^, resulting in collapse and adhesion in the earlier traditional surgical treatment.

The traditional treatment methods include iliac vein adhesion lysis, artificial vascular stent suspension, right iliac artery transection combined with left common iliac vein posterior anastomosis, iliac vein lesions resection and artificial vascular graft *in situ*^2–4^. For patients with iliac vein thrombosis, temporary arteriovenous fistulization combined with suprapubic bypass shunt (Palma) was performed during the surgical thrombectomy. These surgical trauma is large^5^, and the long-term effect is not satisfactory, patients are difficult to accept.

In 1995, Berger *et al*. reported endovascular stent treatment for Cockett syndrome firstly and achieved good curative effect^16^. Subsequently, a number of scholars reported the short-term and medium-term efficacy of endovascular interventional treatment, and concluded that it has good feasibility and safety^17–20^. The author also underwent repair of femoral venous valve for patients with severe blood reflux^6^. For patients with secondary thrombosis, the endovascular technique was applied in the treatment during surgical thrombectomy and the results were satisfactory^21^.

Endovascular interventional treatment of Cockett syndrome should be carried out according to the steps to prevent the occurrence of complications. Firstly, the Seldinger technique must be used to puncture the left femoral vein and successfully insert the catheter sheath^22^, then the guidewire was successfully passed through the diseased iliac vein and inserted into the inferior vena cava with the help of the vertebral artery catheter^23^, followed by the catheter. For patients with complete occlusion of iliac vein, the guidewire should be carefully delivered into the inferior vena cava under the guidance of Roadmap. There are 3 ways to confirm whether the guidewire and catheter enters the inferior vena cava: 1, whether the guidewire can enter the right atrium or enter into the superior vena cava through the right atrium^4^. 2, Catheter was inserted into the inferior vena cava, exit guide wire, pull back to see whether the the blood is refluxing, If the blood was back-flow, the catheter was proved to be in the inferior vena cava^24^, if not, the catheter may not be placed in the inferior vena cava or catheter attached to the lateral wall of the inferior vena cava, spin the tube and pump back. 3, Transcatheter injection of contrast agent, if the inferior vena cava imaging, contrast agent disappeared quickly, it was confirmed that the catheter was placed in the inferior vena cava, if the inferior vena cava does not develop, the contrast agent is detained locally^25^, it indicates that the catheter is not in the inferior vena cava. This method is commonly called “smoking technique”, and the third method “smoking technique” is the most accurate method. In the confirmation of catheter within the inferior vena cava, replace the guide wire with a stiff guide wire, After the guidewire was exchanged, the guidewire should not be moved when the catheter is removed, then the balloon catheter was inserted and balloon angioplasty were performed. The diameter of the balloon should be 12~16 mm, if it is too large, it may cause the rupture of iliac vein. If there is a balloon elastic retraction or residual stenosis after confirmation of the expansion and patients without discomfort, the stent should be implanted. Self expandable stent or Wallstent stent can be chosen, diameter 14~20 mm, the length can completely cover lesions, usually 80~120 mm. This group of 1 cases of patients in technical failure, after the guidewire was inserted into the inferior vena cava and this method verified that, the stiff guide wire also slid out of the inferior vena cava when the vertebral artery catheter exited from the inferior vena cava. The guidewire was inserted into the inferior vena cava again without verification in accordance with the 3 methods mentioned above. The blind introduction of balloon catheter resulting in the super hard wire pierced through the left iliac vein. The dilatation of balloon dilatation catheter further resulted in iatrogenic rupture of the left common iliac vein. The patient suffered from retroperitoneal hemorrhage and hemorrhagic shock.

Therefore, our experience is that the guide wire must be validated for entry into the target vessel through the 3 methods mentioned above especially for the third in the balloon dilatation or stent implantation. Balloon dilation can not be performed until the position of guide wire is determined. In addition, the guidewire must be fixed in the original position when balloon dilation catheter was introduced, otherwise, the position of the guidewire should be verified again. The monitoring of pressure difference before and after operation in group B showed that the pressure difference was significantly decreased (*P* < 0.01), which indicated that the effect was definite.

In this study, the course of disease of patients in group A were less than 7 days, color Doppler ultrasound and/or venography confirmed the presence of blood flow signals or “double track sign” in the lumen and some of them were central thrombosis. The blood flow velocity of the distal vein was normal after thrombectomy. While the distal femoral vein lumen of patients in group B were normal, so the most of them do not need temporary arteriovenous fistula surgery.

Catheter directed thrombolysis (catheter-directed thrombolysis, CDT) is considered to be an effective treatment for deep venous thrombosis^26–28^. Therefore, in the later stage of A group, all patients were treated by catheter-directed thrombolysis technology instead of iliac vein incision thrombectomy, and achieved good results. Anterior tibial vein, posterior tibial vein, popliteal vein and small saphenous vein could be chosen as the approach of thrombolysis catheter during catheter-directed thrombolysis. For some central thrombosis, the ipsilateral femoral vein could be chosen as the blood vessel which thrombolysis catheter enter into, instead of contralateral femoral vein or jugular vein approach^25,29,30^. Because all patients in group A were secondary to deep vein thrombosis on the basis of Cockett syndromehe thrombolysis catheter is difficult to pass through the occlusion or stenosis of the left common iliac vein and is sent into the external iliac vein and femoral vein. And the femoral vein often has valves, the catheter is not easy to pass if forced, it is easy to damage the femoral vein valve which could result in the femoral vein dysfunction.

There are still some difficulties to puncture the anterior tibial vein, posterior tibial vein, popliteal vein and small saphenous vein. In the early stage, we mainly find the target vessel through the operation, In the late stage, with the improvement of endovascular technique and the experience of endovascular treatment of chronic total occlusion lesions in lower extremity arteries, the target vessels were usually punctured under the guidance of the roadmap and/or venography, and the success rate of puncture was over 95%.

In conclusion, combining the result of surgery and follow-up results in our center, we thinks that the endovascular treatment of Cockett syndrome is safe and effective, and it is a good choice for surgical treatment of Cockett syndrome and has good effect in mid-and long-term. For patients with acute iliac vein thrombosis, the combined application of Forgarty catheter embolectomy or catheter-directed thrombolysis is a useful supplement for endovascular treatment. However, this is the single center experience, there is still a need to increase the number of cases, combined with multicenter long-term research and further observation.

### Compliance with Ethical Standards

Ethical approval: This article does not contain any studies with animals performed by any of the authors. All procedures performed in studies involving human participants were in accordance with the ethical standards of the institutional and/or national research committee and with the 1964 Helsinki declaration and its later amendments or comparable ethical standards. The study was approved by the ethics committee of the Second Affiliated Hospital of Nanchang University. Informed consent: Informed consent was obtained from all individual participants included in the study.

### Data availability statement

Materials and Methods are available in the online-only Data Supplement. http://www.yozodcs.com:8000/2017/11/15/MTcxMTE1MDk0NDY1NjYz.html.

## Electronic supplementary material

Dataset 1
