# Supplementary material for: Mid-and long-term efficacy of endovascular-based procedures for Cockett syndrome
Source: Sci Rep. 2018 Aug 14;8:12145. doi: 10.1038/s41598-018-29756-1 (PMC6092402; doi:10.1038/s41598-018-29756-1)
Supplement: Supplementary file 1 — Dataset 1 [file 41598_2018_29756_MOESM1_ESM.doc]

Mid- and long-term efficacy of endovascular-based procedures for Cockett syndrome

Jiasheng Xu^1,2^ Weimin Zhou^1^

**Author unit:**

1. Vascular surgery department, the Second Affiliated Hospital of Nanchang University,
2. Graduate School of Nanchang University

**Postal address:** MINDE ROAD, NANCHANG, 330006, JIANGXI PROVINCE, CHINA

Zip code: 330006

**Correspondent author：**Weimin Zhou Email：jesonxudoctor@163.com

**Ethical review statement**

Approval: The study was approved by the ethics committee of the Second Affiliated Hospital of Nanchang University.

Accordance: Authors of this article solemnly declares that the methods were carried out in accordance with the relevant guidelines and regulations.

Informed consent (for experiments involving humans or human tissue samples):

Informed consent was obtained from all individual participants included in the study. All participants in the study voluntarily signed informed consent.

The study obtained informed consent for the publication of identifying information or images in an online open-access publication（when applicable）.


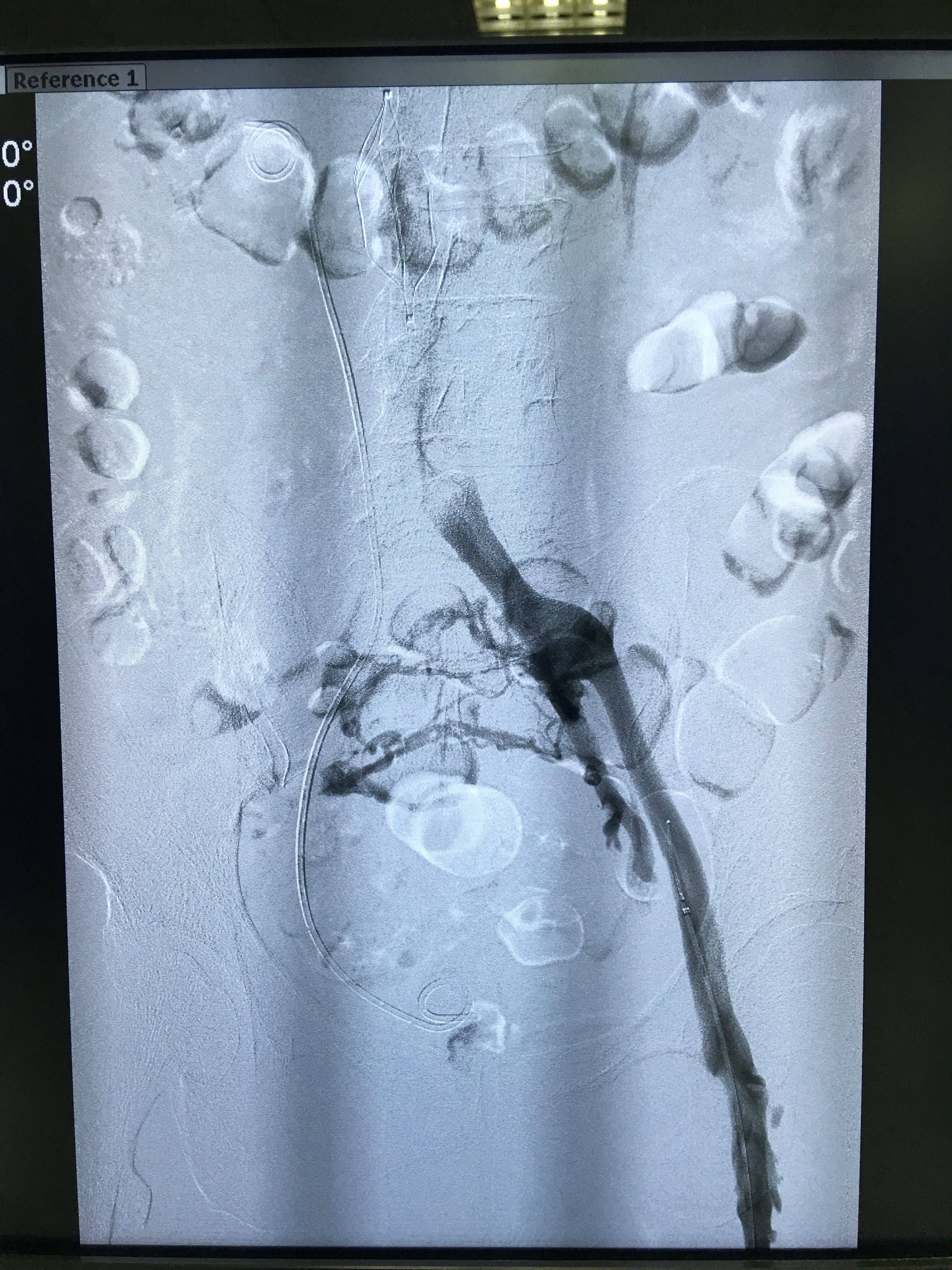


Radiography was performed three days after CDT surgery


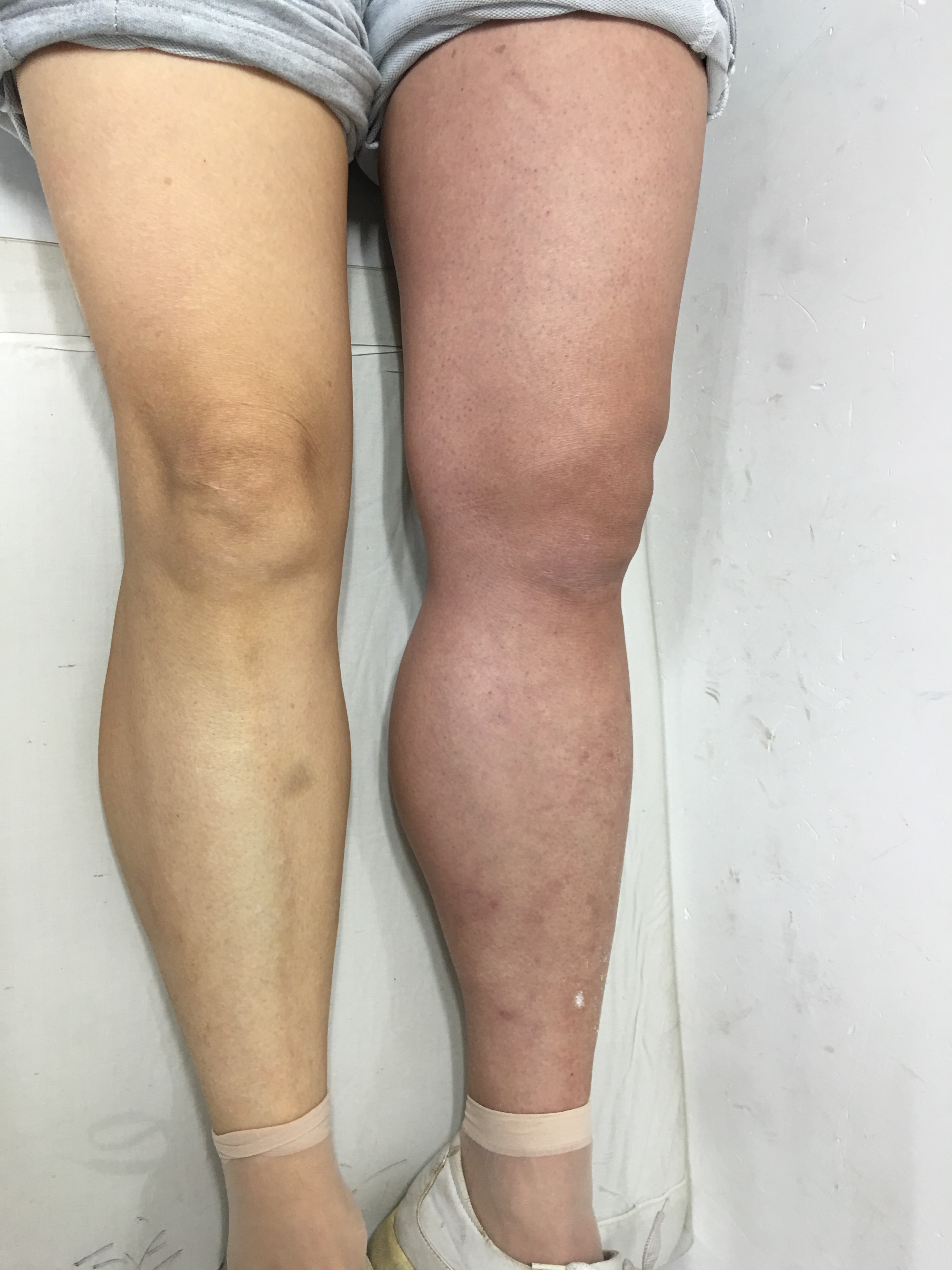


Preoperative condition


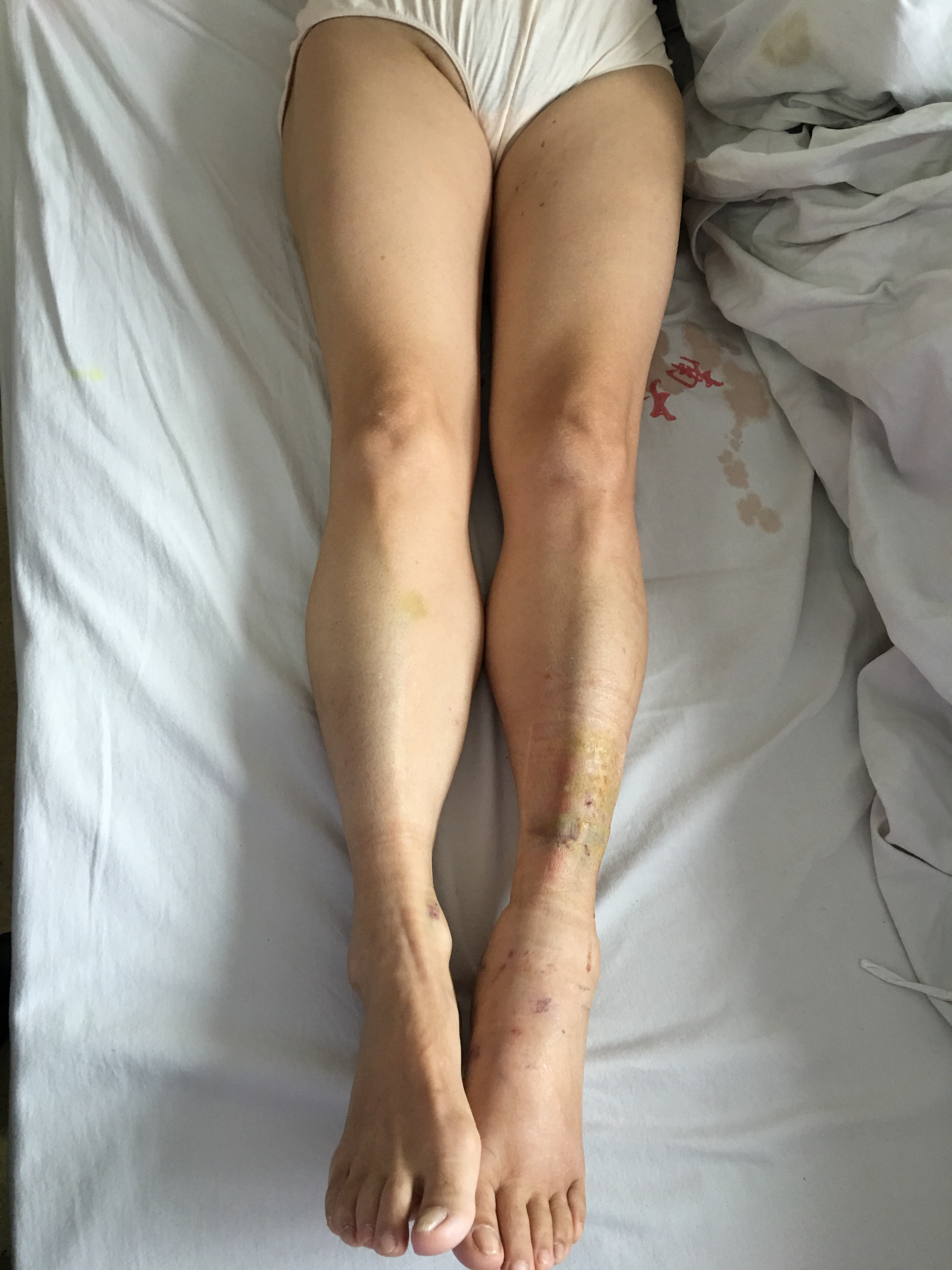


One week after the operation


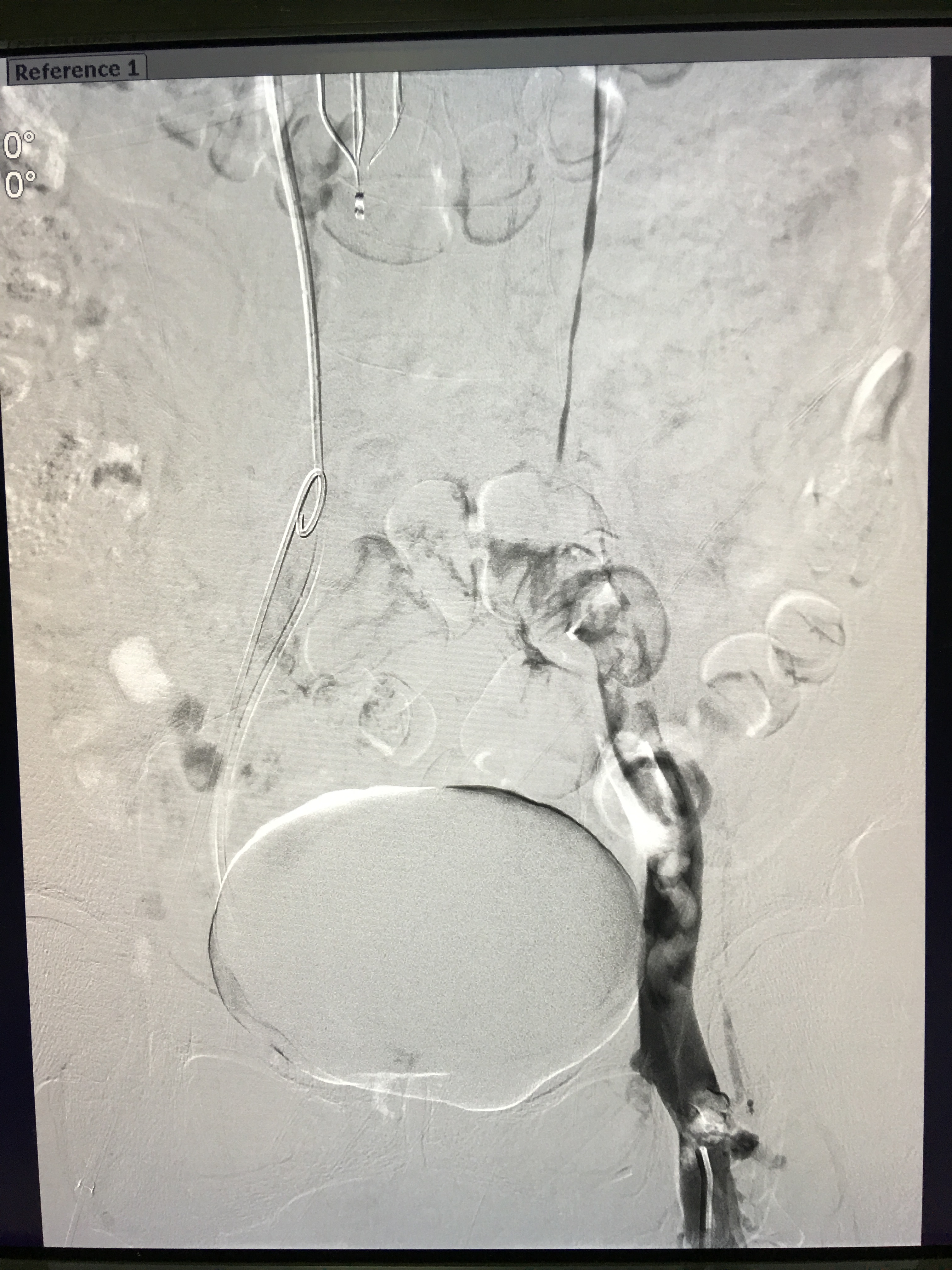


Intraoperative angiography


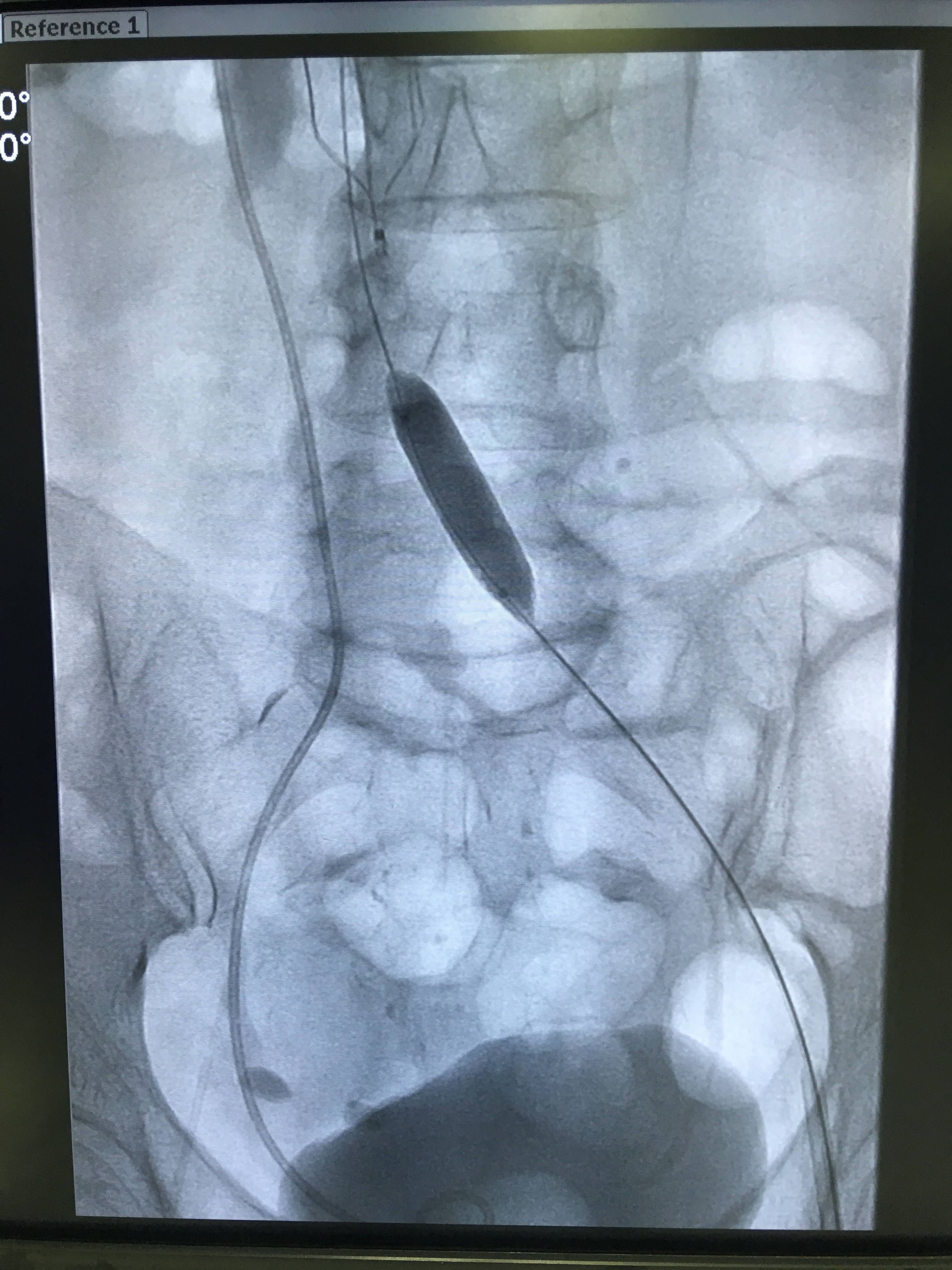


figure 2 C Balloon dilatation of the left liac vein


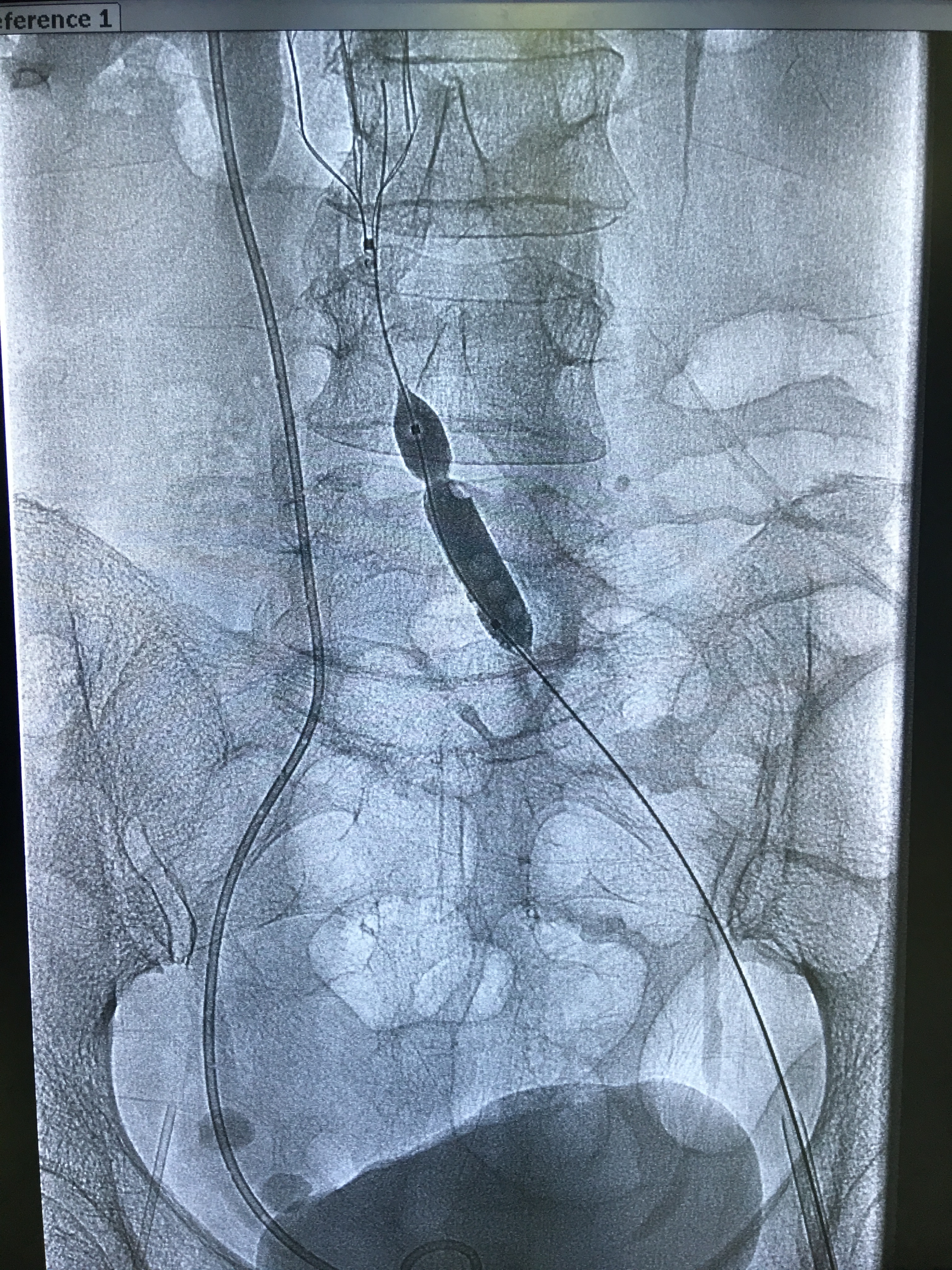


figure 2 B Balloon dilatation of the left liac vein


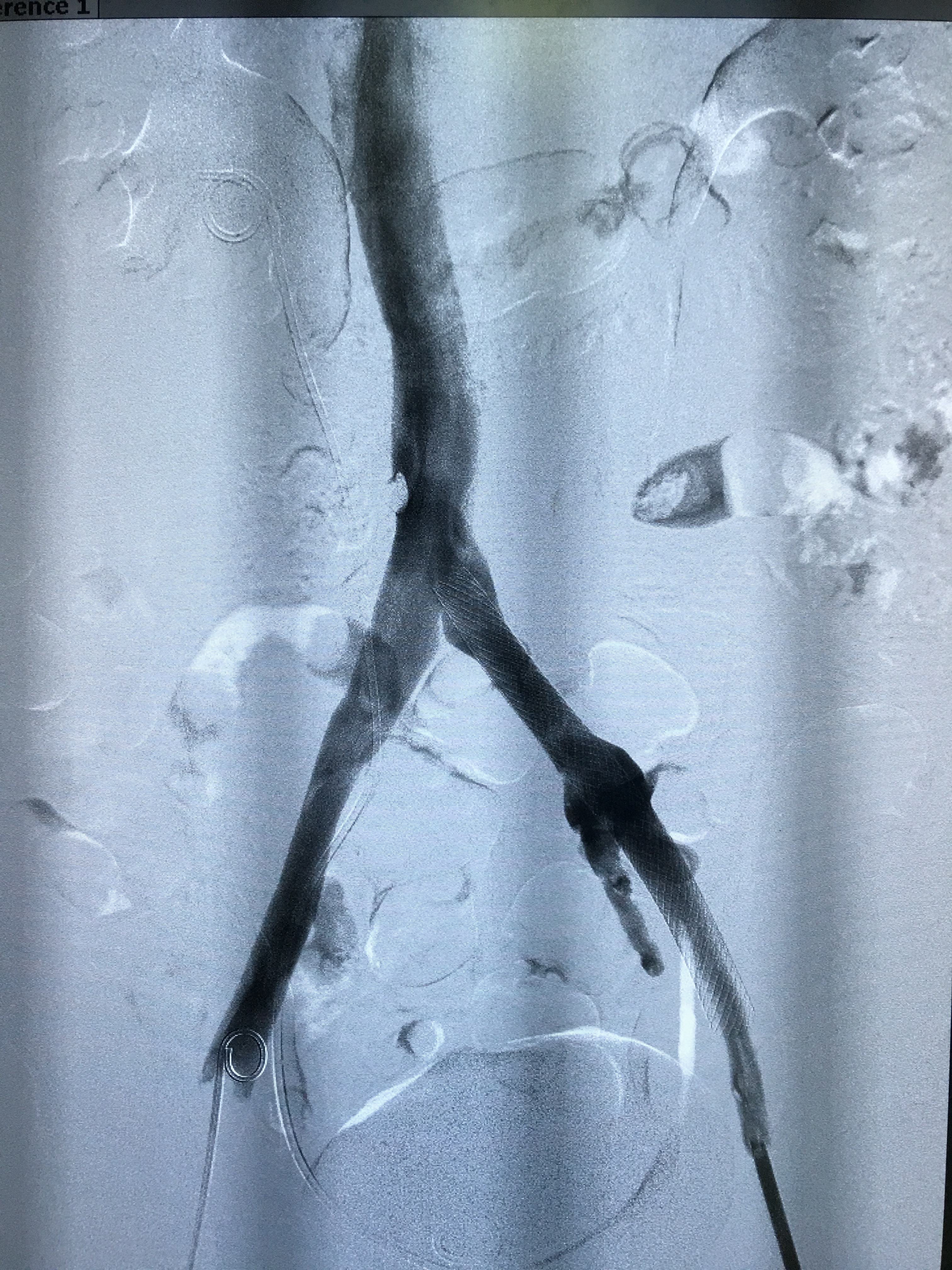


figure 2 A Dissolution of the thrombus in the distal part of the left iliac vein, and presence of the filling defect of the left iliac vein (showed by arrow)


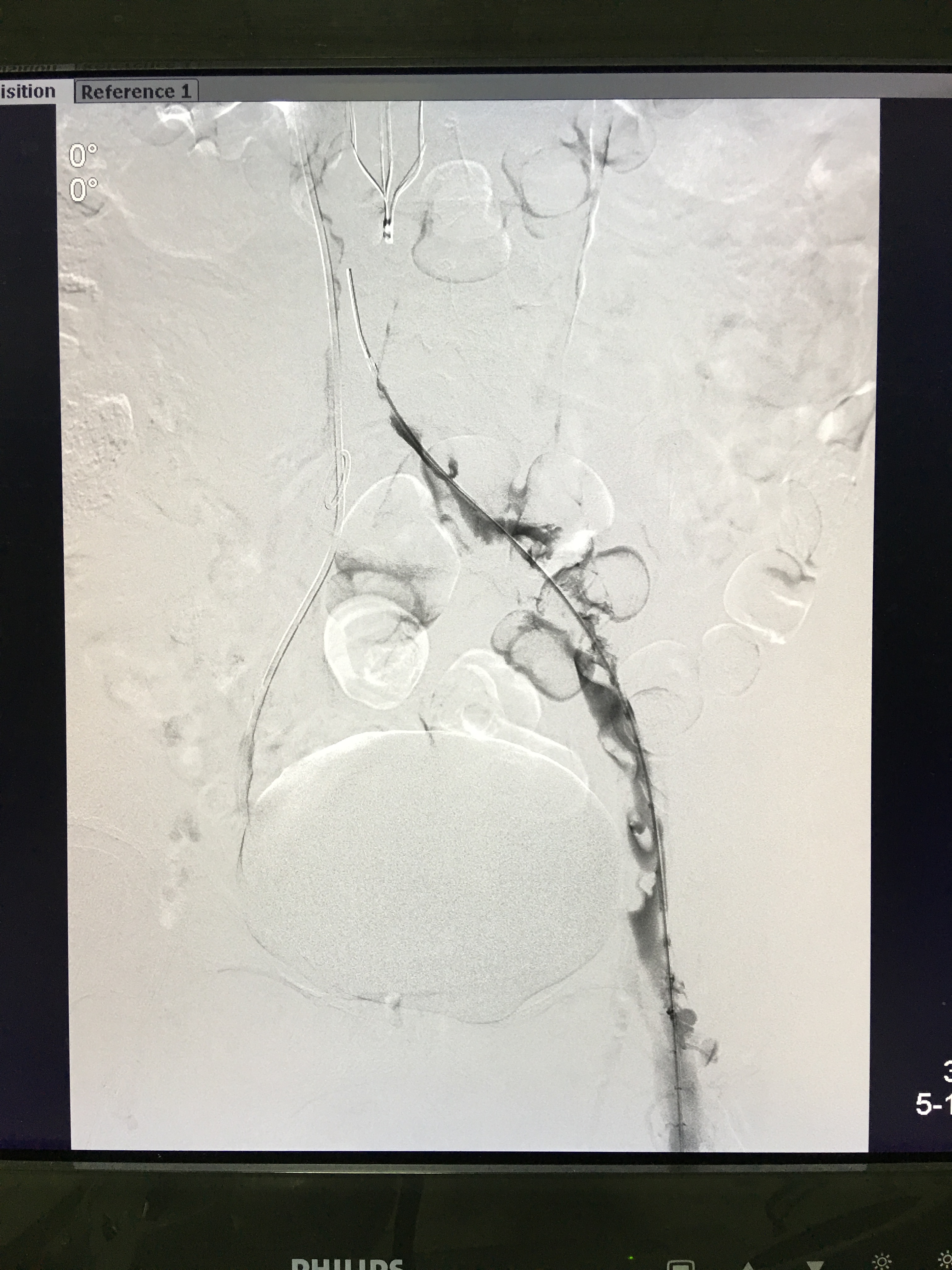


figure 1D 5-F Unifuse catheter placement in the iliofemoral vein


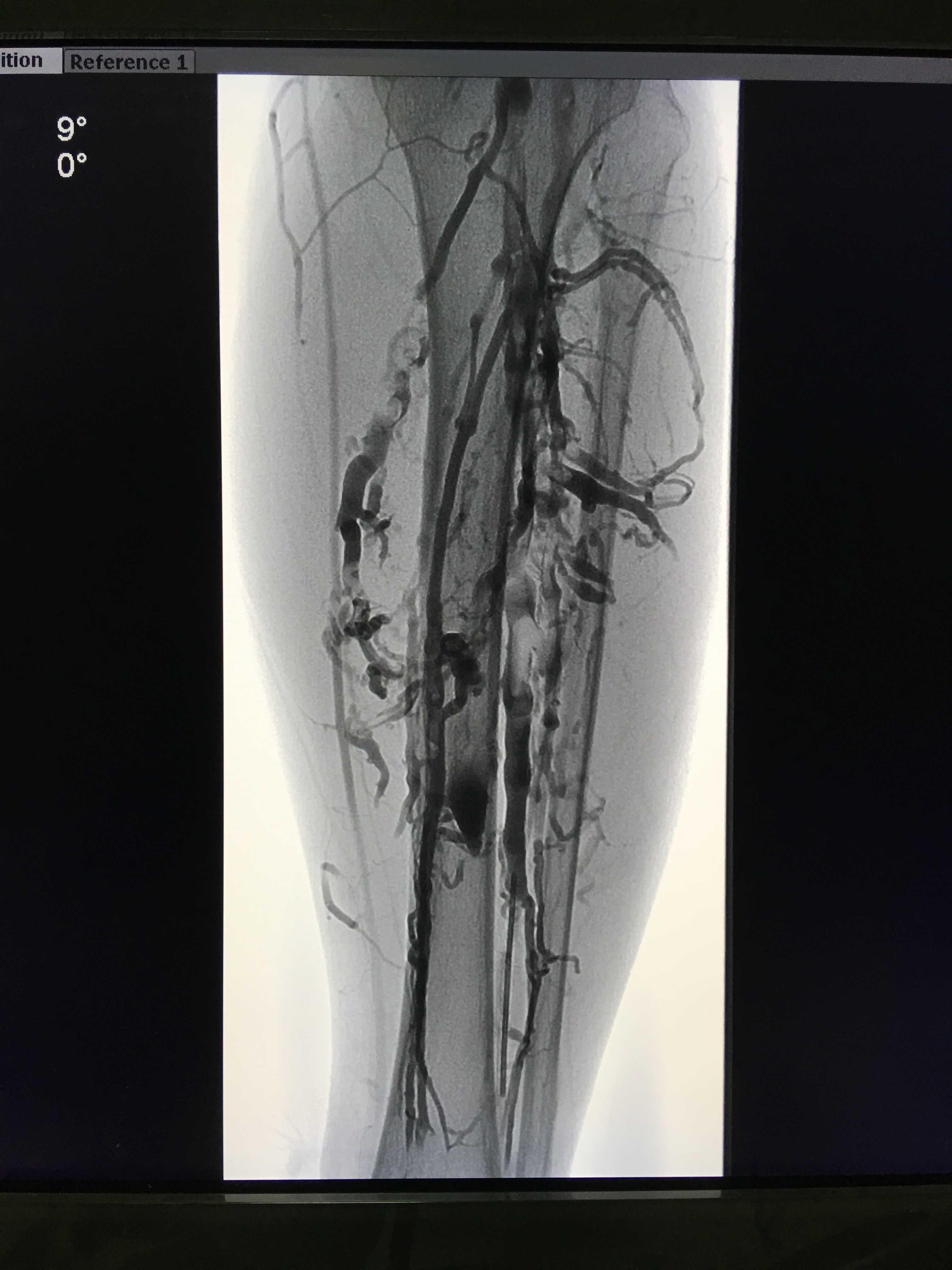


figure 1 C Roadmap anterior tibial vein puncture


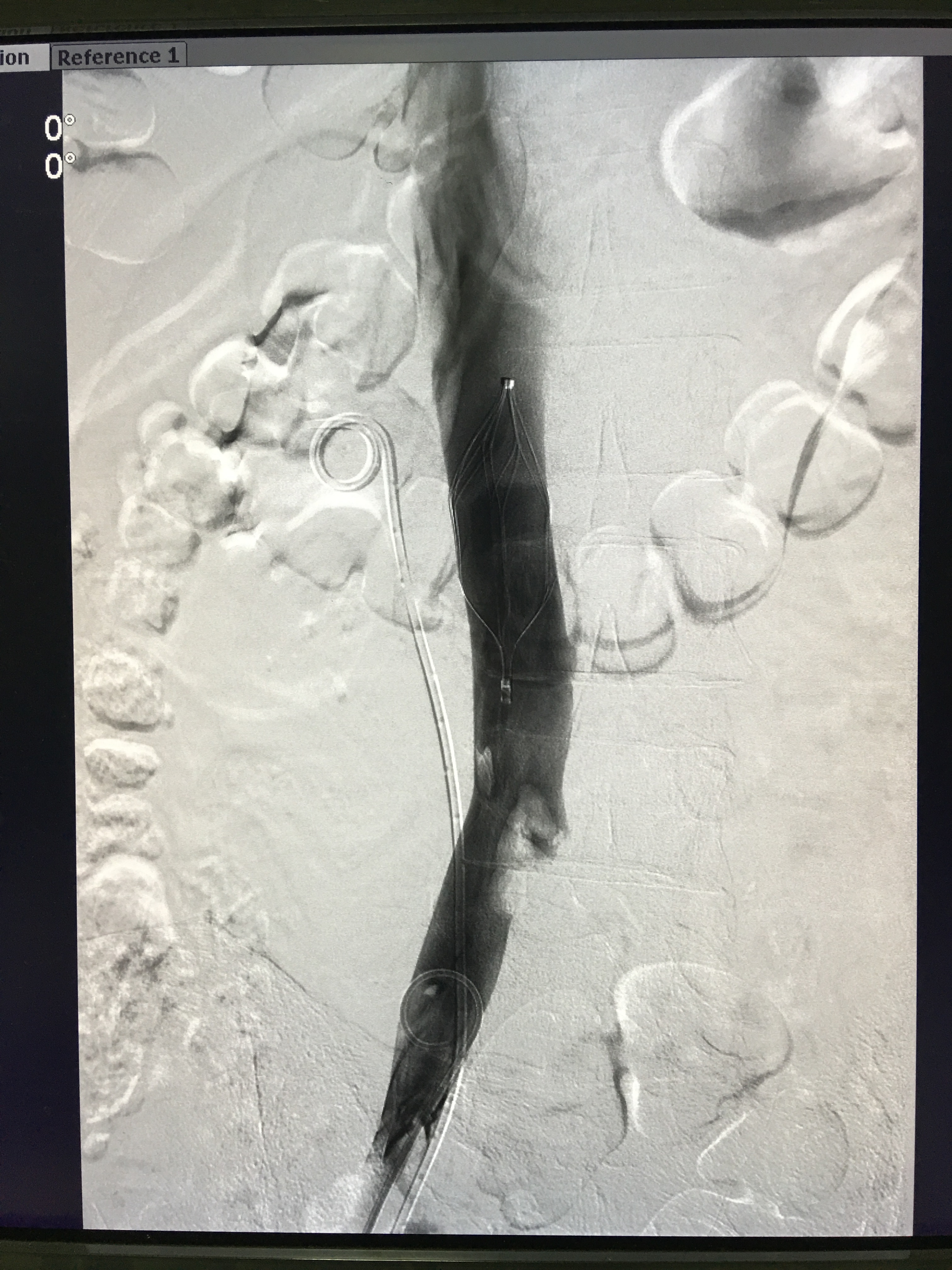


figure 1 B Vena cava filter placement in the inferior vena cava below the renal vein


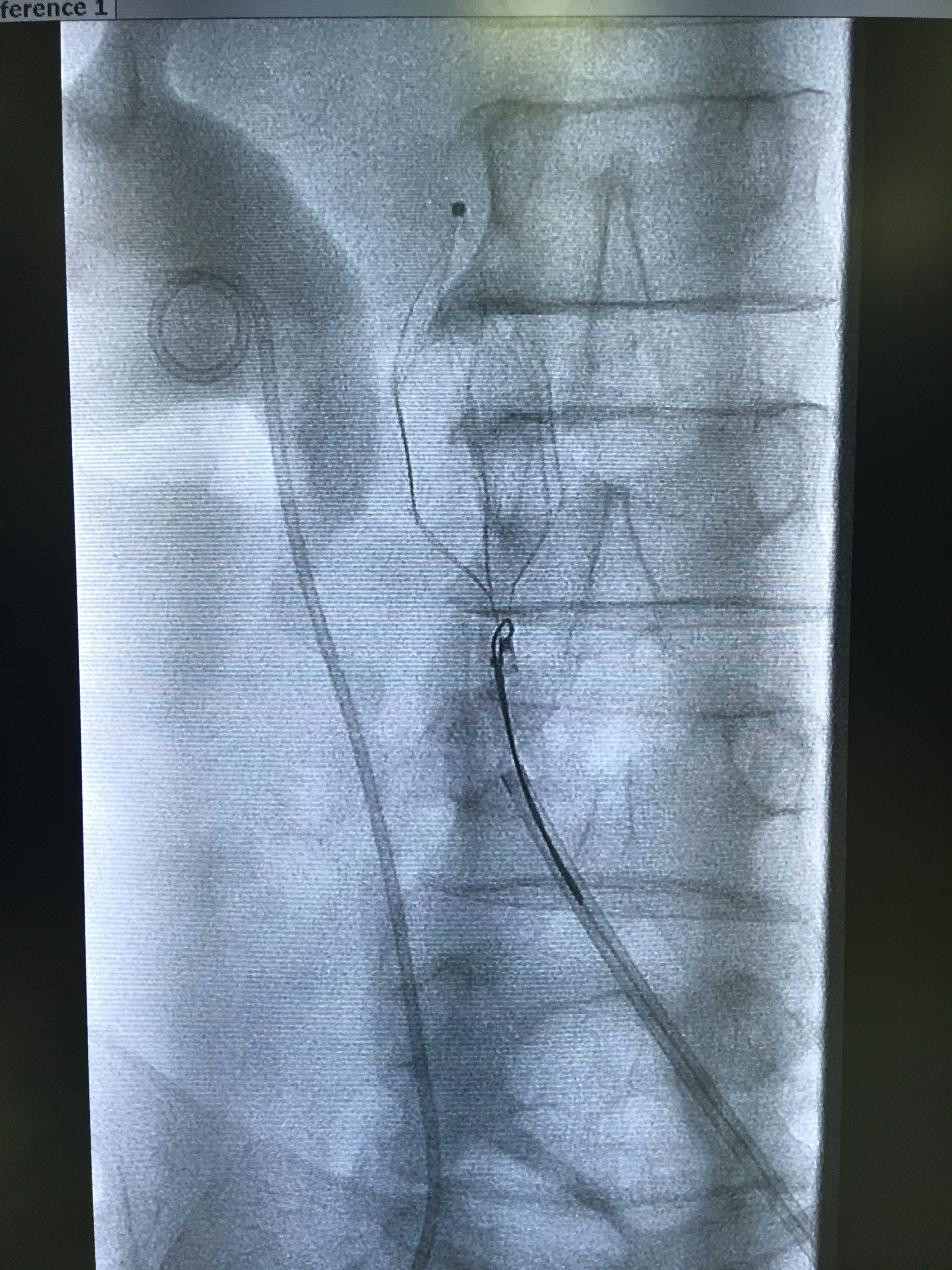


Removal of inferior vena cava filter
